# Multi-channel EEG emotion recognition through residual graph attention neural network

**DOI:** 10.3389/fnins.2023.1135850

**Published:** 2023-07-25

**Authors:** Hao Chao, Yiming Cao, Yongli Liu

**Affiliations:** College of Computer Science and Technology, Henan Polytechnic University, Jiaozuo, China

**Keywords:** EEG, emotion recognition, residual network, graph attention neural network, feature fusion

## Abstract

In this paper, a novel EEG emotion recognition method based on residual graph attention neural network is proposed. The method constructs a three-dimensional sparse feature matrix according to the relative position of electrode channels, and inputs it into the residual network to extract high-level abstract features containing electrode spatial position information. At the same time, the adjacency matrix representing the connection relationship of electrode channels is constructed, and the time-domain features of multi-channel EEG are modeled using graph. Then, the graph attention neural network is utilized to learn the intrinsic connection relationship between EEG channels located in different brain regions from the adjacency matrix and the constructed graph structure data. Finally, the high-level abstract features extracted from the two networks are fused to judge the emotional state. The experiment is carried out on DEAP data set. The experimental results show that the spatial domain information of electrode channels and the intrinsic connection relationship between different channels contain salient information related to emotional state, and the proposed model can effectively fuse these information to improve the performance of multi-channel EEG emotion recognition.

## 1. Introduction

Emotion is a physiological state of human beings accompanied by cognition and consciousness. People's daily cognitive and behavioral activities are almost driven by emotion, which also affects interpersonal interaction and group activities (Guozhen et al., 2016). Affective computing is a representative field, which aims to give computer systems the ability to automatically recognize, understand and respond to human emotions, so as to realize intelligent human-computer interaction. As the core and important component of affective computing, emotion recognition has a wide range of applications in psychology, emotional computing, artificial intelligence, computer vision, medical, and other fields (Ramirez et al., 2001; Hu et al., 2019; Fürbass et al., 2020).

Physiological signals mainly include electrocardiogram (ECG), electromyography (EMG), and electroencephalogram (EEG). Compared with facial expressions and voice signals, physiological signals are not easy to disguise, and are more objective and reliable in capturing the real emotional state of human beings. With the rapid development of wearable devices, long-term monitoring of physiological signals has become a reality, which makes it feasible and practical to judge emotional state based on EEG signals. In the medical field, EEG classification models play a role in automatic diagnosis of psychiatric disorders. Depression is one of the largest health problems in the world. It is a serious mental illness, and there is a problem of untimely treatment. Severe patients often have thoughts of suicide. Current diagnostic criteria for depression are still based on subjective clinical rating scales, such as The Hamilton Depression Rating Scale (Hamilton, 1960), and require physician input (Sung et al., 2005). Some research focuses on automatic diagnosis of depression based on EEG (Alhaj et al., 2011; Mohammadi et al., 2015), which can enable patients to quickly diagnose and understand their own condition, so as to carry out scientific treatment in advance.

The main components of the EEG signal are brain rhythm from different brain regions, reflecting the activity of that region (Niedermeyer and da Silva, 2005). The electrical activity of the cerebral cortex is transmitted to the scalp through the anatomical structure. Therefore, the acquired EEG is a mixture of source signals from different brain regions, carrying a large amount of spatial location information (Xing et al., 2019). In the research field of emotion recognition based on EEG, some studies have explored asymmetric features of brain regions, such as DASM (differential asymmetry), RASM (rational asymmetry), DCAU (differential causality) (Gogna et al., 2016; Li et al., 2018b). And other works studied the connectivity of EEG signals (Nolte et al., 2004, 2008; Supp et al., 2007; Haufe et al., 2013). Castelnovo et al. finds that the electrical activity of the brain is mainly concentrated in specific brain regions when people are in different sleep states, scalp EEG analysis of all night NREM (non-rapid eye movement) sleep revealed a localized decrease in slow wave activity (SWA) power (1–4 Hz) over centro-parietal regions relative to the rest of the brain in SADs compared to good sleeping healthy controls (Castelnovo et al., 2016). Nowadays, there are also some works that make better use of the spatial domain information of EEG channels in EEG classification tasks. In order to learn the spatiotemporal characteristics of EEG signals, Salama et al. divided the original EEG signals into multiple frames, and combined the original EEG signals of multiple channels into a two-dimensional matrix in each frame, where the first dimension represents the number of channels, and the second dimension Indicates the time length of a frame. Multiple frames are then superimposed to form a three-dimensional matrix, with the third dimension representing time. Finally, the 3D matrix is used as the input for 3D-CNN (3d convolutional neural networks) training. Since the left and right hemispheres of the human brain respond asymmetrically to emotion, a bi-hemisphere domain adversarial neural network (BiDANN) model is proposed to learn the discriminative emotional features of each hemisphere, BiDANN contains one global and two local domain discriminators, and learns discriminative sentiment features for each hemisphere by adversarial with local domain discriminators and classifiers (Li et al., 2018c). Li et al. (2017) captures the spatial domain information contained in electrode positions by mapping into EEG multidimensional feature image following a 10/20 system. First, the spatial features, frequency domain and time features of the EEG signal are integrated, and mapped into a feature matrix according to the international 10/20 system, and then the EEG multidimensional feature image is generated using the interpolation method, using a combination of convolutional neural network (CNN) and long-term and short-term A hybrid deep network of memory (LSTM) recurrent neural network (RNN) recognizes emotional states. Li et al. (2018a) also used the distribution of electrodes on the scalp to extract the spatial domain information of electrode locations. First, the differential entropy features from 62 EEG signal channels are organized into a two-dimensional map of 8×9, and are mapped to a 20×20 input map through sparse operations to avoid information leakage in convolution and pooling operations. Finally, hierarchical convolutional neural network (HCNN) is used to classify positive, neutral and negative emotional states.

To a certain extent, the above research has applied the extraction of the spatial domain information of the EEG channel, and used the multi-dimensional feature matrix mapped according to the international 10/20 system and CNN to fuse the information of the neighbor nodes. However, there still exist several challenges in multi-channel EEG-based emotion recognition. First of all, the brain activity in emotional state is complex, and multiple brain regions are involved in the interaction. How to effectively characterize the interaction between brain regions is a problem to be considered. Furthermore, due to the local perception characteristics, CNN (Convolutional Neural Networks) tends to pay more attention to adjacent electrode channels and is good at learning local spatial patterns. Therefore, in the process of extracting electrode spatial position information, CNN can mine the significant information of correlation and interaction of different EEG signals in the same brain region. However, it cannot effectively capture the intrinsic connection relationship between EEG channels located in different brain regions and the global spatial position information of electrodes. Finally, the features extracted from the EEG signal and the distance between different electrodes are a kind of non-Euclidean data, only mapping the features extracted from each channel into a multi-dimensional sparse feature matrix according to the international 10/20 system ignores the distance information between electrodes, and ignores that all electrodes are not positioned in an absolute plane on the scalp.

To solve the above problems, this paper proposes a noval emotion recognition method based on residual graph attention neural network (ResGAT). In the proposed method, the residual network is utilized to achieve the spatial position information of the electrode channel and the correlation information of the adjacent EEG channels through the 3D feature matrix. Considering that the graph neural network (GAT) can update the state of vertices by periodically exchanging neighborhood information without being limited by vertex distance, it is employed to learn the neural functional connections between different brain regions, and the multi-head self-attention mechanism is used to adaptively adjust the adjacency matrix in the network. Therefore, the ResGAT model makes full use of the electrode spatial position information and the intrinsic connection relationship between EEG channels located in different brain regions. Moreover, when the EEG channel aggregates the characteristics of neighboring nodes, it pays more attention to the channel that is more relevant to itself. Finally, the high-level abstract features representing electrode space domain information and the high-level abstract features representing intrinsic connection relationship between EEG channels located in different brain regions are fused to judge the emotional state.

## 2. Datasets and feature extraction

### 2.1. Data set

The DEAP data set used in the experiment is an open data set collected through experiments by Koelstra et al. from Queen Mary University of London, University of Twente, University of Geneva, Switzerland, and Swiss federal Institute of Technology in Lausanne to analyze human emotional states (Koelstra et al., 2011). The dataset records multimodal physiological signals of 32 volunteers under the stimulation of selected music videos, including EEG and peripheral physiological signals, and 22 of the 32 volunteers also record facial expression videos. Each volunteer needs to watch 40 1-min long videos using 32 active AgCl electrodes (Fp1, AF3, F3, F7, FC5, FC1, C3, T7, CP5, CP1, P3, P7, PO3, O1, Oz, Pz, Fp2, AF4, Fz, F4, F8, FC6, FC2, Cz, C4, T8, CP6, CP2, P4, P8, PO4, and O2) recording EEG signals, these electrodes were placed on the scalp according to the international 10/20 system. At the end of each trial, the valence, arousal, dominance, and liking of the video were evaluated on a scale of 0–9. Physiological signals were sampled at 512 Hz and resampled at 128 Hz. The physiological signal matrix of each subject is 40×40×8064 (40 trials, 40 channels, 8,064 sampling points). Eighty thousand and sixty-four is 63 s data at 128 Hz sampling rate, 3 s silent time.

In addition, the effectiveness of ResGAT was verified using the SEED-IV brain emotion dataset (Zheng et al., 2018). This data set selected 72 movie clips containing 4 emotions (Happy, Sad, Neutral, and Fear) as EEG-induced materials. A total of 15 subjects recorded 62-channel EEG signals and eye movements when watching movie clips.

### 2.2. Feature extraction

In the DEAP set, each person watched 40 emotion-inducing videos, and the duration of EEG signals recorded in each video was 60 s. In the experiment, a sliding window divides the raw EEG signal of each channel into several segments, and the duration of each sliding window is set to 6 s. The segments do not over lap. Each segment is considered an independent sample, and the six new samples inherited the labels of the original. Thus, 12,800 samples were be obtained. A set of time domain features can be extracted from the 32-channel EEG signals of each sample, specifically including mean, variance, first difference value, second difference value, standard deviation, and fuzzy entropy. Among them, Fuzzy Entropy was proposed by Chen et al. (2007) and applied to the representation of EMG signals. Fuzzy Entropy introduces the concept of fuzzy sets. Based on the exponential function and its shape, the similarity of vectors is vaguely defined in FuzzyEn, compared with ApEn and SampEn, the FuzzyEn is an effective measure algorithm for analyzing chaotic sequence complexity, it has better robustness and measure value continuities.

The soft continuous boundary of the fuzzy function ensures the continuity and effectiveness of the fuzzy entropy under small parameters, so the more details obtained by the fuzzy function also make the fuzzy entropy a more accurate definition of entropy. Assuming that the EEG signal of each channel is represented by s(T), t = 1, 2..., T, T is the signal length, which is 128×60 (frequency×second), the measure of the length of the EEG signal subsequence in fuzzy entropy m is 2. By reconstructing the original sequence, we can get


(1) $$\begin{array}{l} X_{i}^{m}=\left\{s\left(i\right),s\left(i+1\right),...,s\left(i+m-1\right)\right\}-s_{0}\left(i\right) \end{array}$$

Among them, i=1,2,...,N-m+1. $X_{i}^{m}$ represents m consecutive s values, *s*_0_(*i*) represents the average value, calculated as follows,


(2) $$\begin{array}{l} s_{0}\left(i\right)=\frac{\sum_{j=0}^{m-1}s\left(i+j\right)}{m} \end{array}$$

Define the maximum difference $d_{ij}^{m}$ between elements in two m-dimensional vectors $X_{i}^{m}$ and $X_{j}^{m}$ as the distance between them,


(3) $$\begin{array}{l} d_{ij}^{m}=\underset{k\in \left(0,m-1\right)}{max}\left\{|s\left(i+j\right)-s_{0}\left(j\right)-\left(s\left(i+k\right)-s_{0}\left(i\right)\right)|\right\} \end{array}$$

The similarity between $X_{i}^{m}$ and $X_{j}^{m}$ can be defined by a fuzzy function,


(4) $$\begin{array}{l} D_{ij}^{m}=\mu \left(d_{ij}^{m},r\right) \end{array}$$

Structure φ_*m*_(*r*) and φ_*m*+1_(*r*),


(5) $$\begin{array}{l} \varphi_{m}\left(r\right)={\left(N-m\right)}_{-1}\overset{N-m}{\underset{i=1}{\sum }}\phi_{i}^{m}\left(r\right) \end{array}$$

(6) $$\begin{array}{l} \varphi_{m+1}\left(r\right)={\left(N-m\right)}_{-1}\overset{N-m}{\underset{i=1}{\sum }}\phi_{i}^{m+1}\left(r\right) \end{array}$$

Then can define the parameter *FuzzyEn*(*m, r*) of the time series as:


(7) $$\begin{array}{l} FuzzyEn\left(m,r\right)=\underset{x\to -\infty }{\lim}\left[\ln\varphi^{m}\left(r\right)-\ln\varphi^{m+1}\left(r\right)\right] \end{array}$$

Among them, when N is finite, it can be estimated by statistics,


(8) $$\begin{array}{l} FuzzyEn\left(m,r,N\right)=\ln\varphi^{m}\left(r\right)-\ln\varphi^{m+1}\left(r\right) \end{array}$$

Two emotion dimensions are used in the experiments. For each sample, if the self-assessment value of the arousal is greater than 5, the category label of the sample is set as the high arousal (HA), otherwise it is set as the low arousal (LA). In the valence-sentiment dimension, the same label division is used for samples, including the high valence (HV) and the low valence (LV).

## 3. ResGAT emotion recognition framework

**Figure 1:**
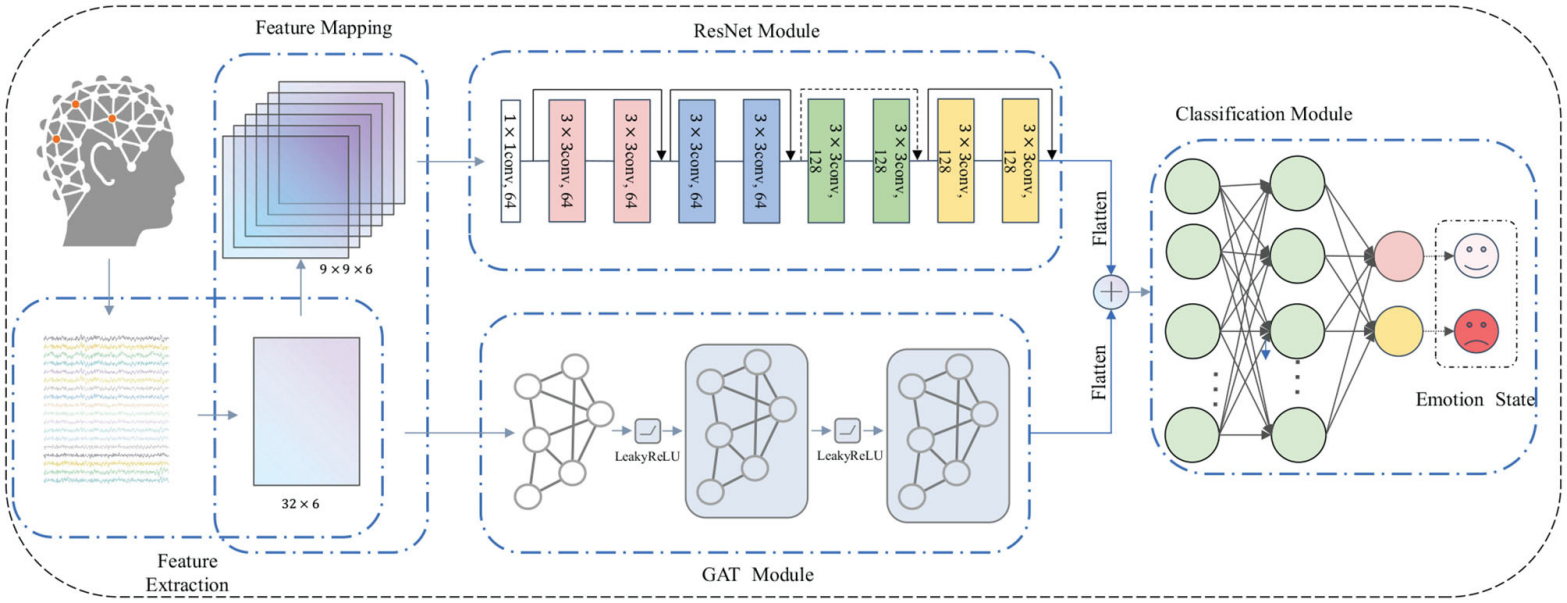
ResGAT emotion recognition framework.

The structure of the proposed ResGAT model is described in Figure 1. The framework includes feature extraction and feature mapping module, ResNet modules, GAT modules and Classification modules. The first part is feature extraction and feature mapping modules, which extracts 6 kinds of temporal features from 32 EEG signals. Then, a 2D electrode position mapping matrix and a 3D sparse feature matrixand are constructed according to the temporal features. The 2D electrode position mapping matrix is input into the GAT modules to extract high-level abstract features, which contain the intrinsic connection relationships between EEG channels located in different brain regions. The ResNet modules is employed to receive the 3D sparse feature matrix and generate high-level abstract features representing electrode spatial position information. Finally, the classification modules is utilized to fuse the two high-level abstract features and judge the emotional state.

### 3.1. Extraction of spatial domain information based on ResNet Module

**Figure 2:**
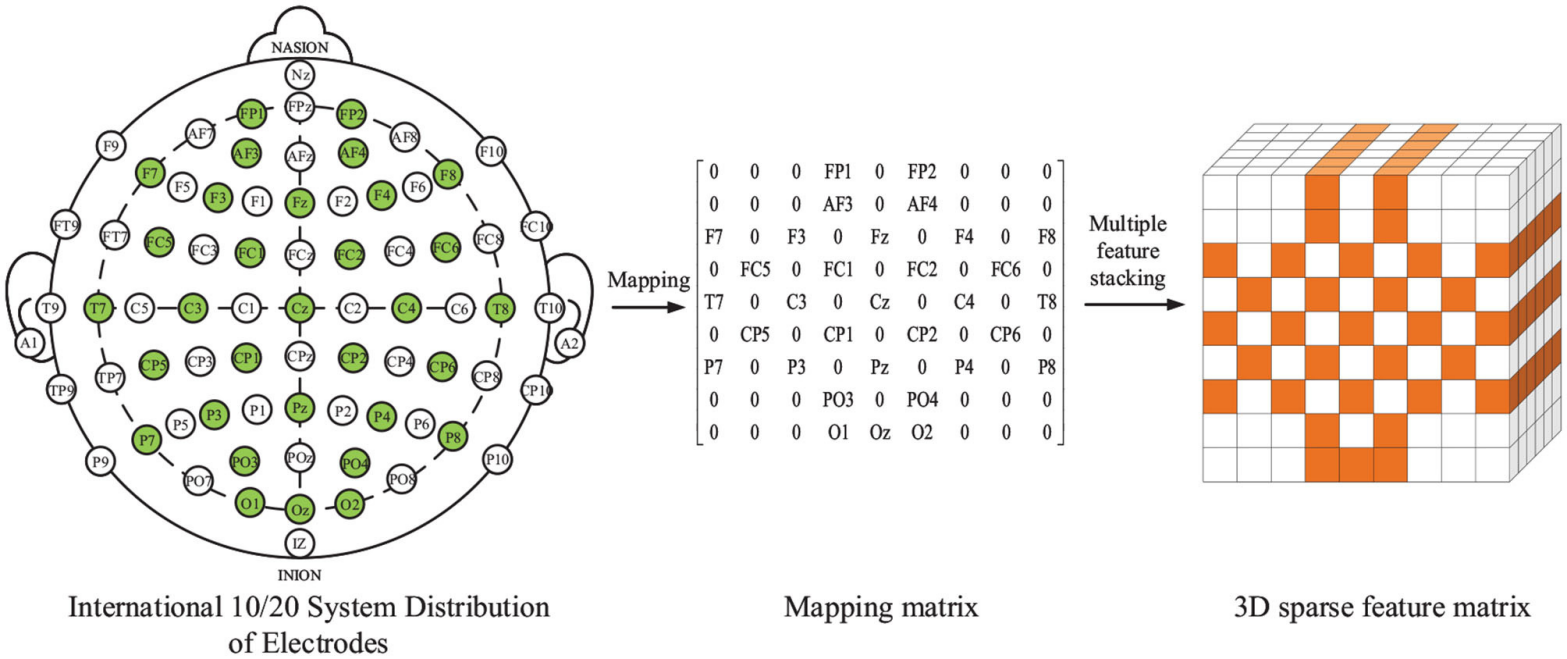
International 10/20 system, 9×9 mapping matrix and 3D sparse feature matrix.

Figure 2 shows the international 10/20 system plan, 2D electrode position mapping matrix and 3D sparse feature matrix *X*^*R*^ ∈ ℝ^*h*×*w*×*c*^. The values of parameter h and parameter w are both set to 9, and the value of parameter c is 6, indicating that the shape of the 3D feature matrix is. The left side of Figure 2 shows the International 10/20 system, where the EEG electrodes marked by green circles are the test points used in the DEAP dataset. Some researches (Li et al., 2017; Chao and Dong, 2020; Cui et al., 2020) have found that spatial features of EEG channels can improve the performance of emotion recognition. In order to represent the spatial location information of all EEG signal channels, a feature matrix is constructed according to the positions of electrodes on the brain, and the spatial parts of different EEG signal channels are mined. In the feature matrix, the time-domain features extracted from different EEG channels are put into the corresponding positions in the matrix by name, and the positions of unused electrodes in the matrix are set to 0. Finally, a 9×9×6 three-dimensional feature matrix is constructed according to the six extracted features, as shown in the right of Figure 2. For each 9×9×1 matrix, different time domains are arranged according to the mapping rules shown in Figure 2 mapping matrix. Finally, the extracted 3D sparse feature matrix is represented by *X*^*R*^, *h* = *w* = 9, *c* = 6, indicating that the shape of the 3D feature matrix is 9×9×6.

After constructing the three-dimensional sparse mapping matrix, it is input into the residual network to extract high-level abstract features. The residual network structure adopted is shown in Figure 1. It is composed of multiple residual blocks. Each residual block is composed of multiple convolution layers, batch normalization layers and activation layers. The size of the convolution kernels used in this part is 3×3. The residual block is calculated as follows:

First, the 3D sparse feature map *U* ∈ ℝ^*h*′×*w*′×*c*′^ is obtained from *X*^*R*^ ∈ ℝ^*h*×*w*×*c*^ by transforming *F*_*tr*_. For transform *F*_*tr*_, it is a Convolution operation. Use $V=\left[v_{1},v_{2},...,v_{c^{\prime }}\right]$ to represent the filter set, where *v*_*i*_ refers to the parameter of the *i*_*th*_ filter. The output is $U=\left[u_{1},u_{2},...,u_{c^{\prime }}\right]$, and


(9) $$\begin{array}{l} u_{i}=v_{i}*X^{R} \end{array}$$

Here, * means convolution, the filter can learn the spatial position information of electrodes and the interaction information between electrodes in local spatial position through convolution operation.

The normalized network response after batching is $Z=BN\left(U\right)=\left[z_{1},z_{2},...,z_{c^{\prime }}\right]$.

Batch normalization can effectively prevent the gradient explosion and gradient disappearance in the network, and speed up the convergence speed of the network. Finally, the nonlinear interaction between the feature map channels is learned through the activation layer, and the complete dependence between the channels is obtained. It is expressed by the following formula:


(10) $$\begin{array}{l} S=WZ,W\in {\mathbb{R}}^{c^{\prime }} \end{array}$$

Among them, δ refers to the relu activation function. After multiple convolutions and activation calculations, the final EEG signal characteristics are expressed as $S^{R}=\left[s_{1},s_{2},...,s_{c^{\prime }}\right]$.

### 3.2. Dynamic learning of the intrinsic connection relationship between EEG channels located in different brain regions

**Figure 3:**
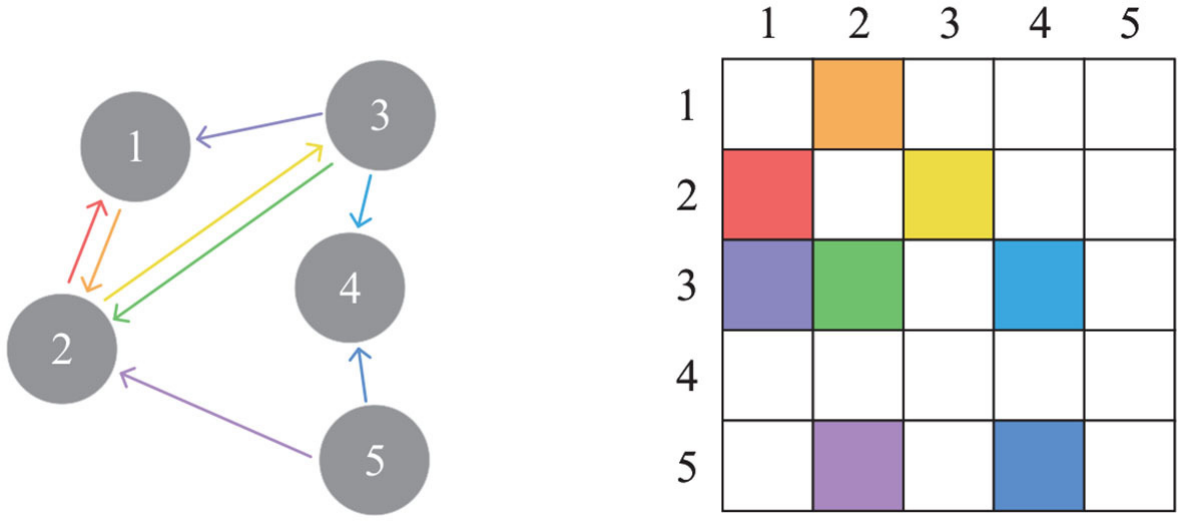
A directed graph and the corresponding adjacency matrix.

As the basis of ResGAT method, some basic knowledge about graph representation is introduced first. A directed connected graph can be defined as *G* = *V, E, W*, where *V* represents the node set with the number of |*V*| = *N*, and *E* represents the edge set connecting these nodes. Let *W* ∈ ℝ^*N*×*N*^ represents the adjacency matrix describing the connection between any two nodes in *V*, in which the entry of *W* in row *i* and column *j* measures the importance of the connection between node *i* and *j*. Figure 3 shows five nodes and edges connecting those nodes, as well as the adjacency matrix associated with the graph. The different colored arrows on the left side of the figure represent the edges connecting the source node and the target node, while the corresponding adjacency matrix is on the right side of the figure.

In the past, convolutional neural networks have been applied in many fields due to their powerful modeling capabilities, such as computer vision, speech recognition, and natural language processing. Due to its locality and translation invariance properties, it is very suitable for processing Euclidean data.However, many elements in the real world exist in the form of graph data, such as social networks, transportation networks, and drug discovery.The features extracted from the EEG signal and the distance between different electrodes are non-European data. Although the number of features on each signal channel is consistent, the distance between each adjacent electrode is uneven, and brain functional connectivity tends to capture global relationships among EEG channels. Therefore, the graph neural network is more suitable for learning the potential internal connections between different channels.At present, the graph attention network (GAT) (Veličković et al., 2017) is a widely used graph neural network. GAT achieves information aggregation in the spatial domain by introducing an attention mechanism, making the model pay more attention to the mutual influence between neighbor nodes, and applying it to EEG data to make the channels aggregate the characteristics of neighbor nodes and pay more attention to channels that are more relevant to themselves. Each EEG electrode can be regarded as a node of the graph, and the connection between the electrodes corresponds to the edge of the graph. The weights of all edges, which representing the functional relationship between electrodes, constitute the adjacency matrix of the graph. Therefore, GAT can learn the internal relationship between different EEG electrodes. As shown in the attention neural network in Figure 1, although GAT can describe the connection between different nodes according to their spatial positions, the connection between EEG channels should be determined in advance before applying it to the construction of emotion recognition model. In addition, it should be noted that the spatial location connection between EEG channels is different from the functional connection between them. In other words, closer spatial relationships may not guarantee closer functional relationships.

The flow of processing EEG signal features with GAT is shown in Figure 1. After data acquisition, preprocessing and feature extraction, EEG data are represented by undirected graph *G* = *V, E, W*. The data on can be represented as feature matrix *X*^*G*^ ∈ ℝ^*n*×*d*^, where *n* represents the number of electrodes and *d* represents the number of features extracted on each electrode channel. The constructed initial adjacency matrix *W*^*G*^ ∈ ℝ^*n*×*n*^, where *n* represents the number of electrode channels, characterizes the correlation between 2D space electrodes. Assume that each electrode channel has an internal relationship with the other 31 electrode channels, and is initialized as a diagonal matrix with the main diagonal of 0 and other values of 1. The feature combination extracted from each EEG channel is represented as a node in the graph neural network model, can be expressed as:


(11) $$\begin{array}{l} X^{G}=\overset{\to }{x_{1}},\overset{\to }{x_{2}},...,\overset{\to }{x_{n}},\overset{\to }{x_{i}}\in {\mathbb{R}}^{d} \end{array}$$

In order to obtain sufficient expression ability to transform input features into higher-level features, at least one learnable linear transformation is needed. A shared *H* ∈ ℝ^*d*′×*d*^ applies to all nodes to increase the expression ability of node features.

Then, a self attention mechanism is used on all nodes. At this time, the dimension of features on the nodes remains unchanged, which is ℝ^*d*′^. The self attention mechanism is described as:


(12) $$\begin{array}{l} e_{ij}=Att\left(H\overset{\to }{x_{i}},H\overset{\to }{x_{j}}\right) \end{array}$$

where *Att* stands for self attention mechanism, and *e*_*ij*_ represents the importance of the characteristics of node *j* to *i*. Only the first-order neighbors of each node is calculated. In order to make the coefficients easy to compare between different nodes, the softmax function is used to normalize the attention coefficients of node *j* to other neighbor nodes.


(13) $$\begin{array}{l} a_{ij}=softmax\left(e_{ij}\right)=\frac{exp\left(e_{ij}\right)}{\sum_{k\in N_{i}}exp\left(e_{ik}\right)} \end{array}$$

where *a*_*ij*_ is the coefficient of attention mechanism. In fact, the attention mechanism a is composed of a single-layer feedforward neural network, and the leakyrelu activation function is used for non-linear processing. Finally, the coefficient of attention mechanism can be expressed as:


(14) $$\begin{array}{l} a_{ij}=\frac{exp\left(e_{ij}\right)}{\sum_{k\in N_{i}}exp\left(e_{ik}\right)}\\ \;=\frac{exp\left(LeakyReLU\left(\overset{\to }{a^{T}}\left[H\overset{\to }{x^{i}}∥H\overset{\to }{x^{j}}\right]\right)\right)}{\sum_{k=1}^{k\in N_{i}}exp\left(exp\left(LeakyReLU\left(\overset{\to }{a^{T}}\left[H\overset{\to }{x^{i}}∥H\overset{\to }{x^{j}}\right]\right)\right)\right)},\overset{\to }{a}\in {\mathbb{R}}^{2d}\; \end{array}$$

where ∥ indicates connection operation.

Then apply the normalized attention coefficient to the features corresponding to the node, and get the output after feature recalibration:


(15) $$\begin{array}{l} \overset{\to }{x_{i}^{\prime }}=\sigma \left(\underset{j\in N_{i}}{\sum }a_{ij}H\overset{\to }{x_{j}}\right) \end{array}$$

Veličković et al. (2017) found that it is beneficial to use multi head attention mechanism in graph neural network. Using *K* independent attention mechanisms at the same time, Formula 14 will produce *K* outputs. Then splice the above *K* outputs together, as shown in the following formula:


(16) $$\begin{array}{l} \overset{\to }{x_{i}^{\prime }}={∥}_{k=1}^{K}\sigma \left(\underset{j\in N_{i}}{\sum }a_{ij}^{k}H^{k}\overset{\to }{x_{j}}\right) \end{array}$$

The output of each node changes to *Kd*′. In the experiment, the *K* is 2.

**Figure 4:**
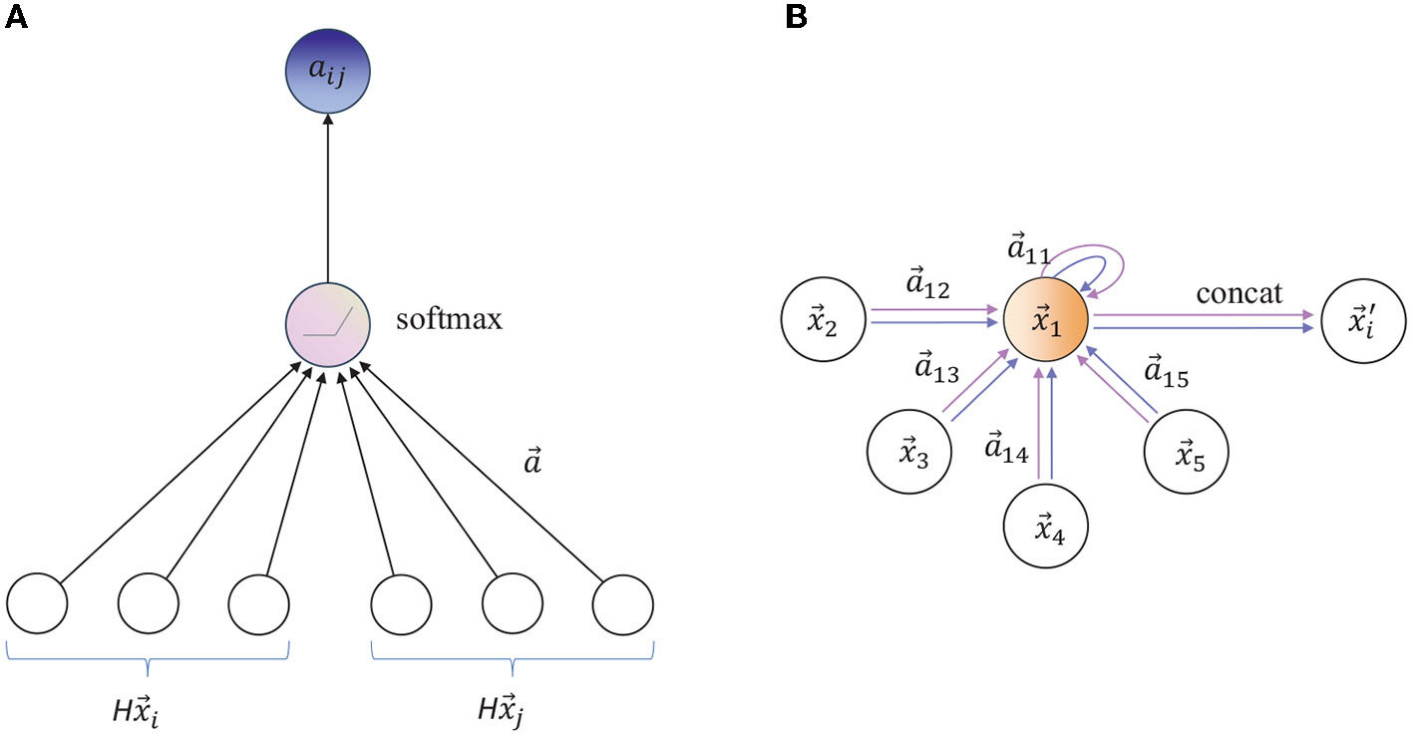
**(A)** The attention mechanism $a\left(H\overset{\to }{x_{i}},H\overset{\to }{x_{j}}\right)$ is parameterized by the weight vector *a* ∈ ℝ^2*d*′^. **(B)** Illustration of multi headed attention (k = 2) of node 1 in its neighborhood. Arrows of different colors indicate independent attention calculation. Aggregate features from each head are connected to obtain $\overset{\to }{x_{i}^{\prime }}$.

The aggregation process of multi head attention mechanism on nodes is shown in Figure 4.

The above is a complete graph convolution process. After multi-layer graph convolution, the EEG features will be further transmitted to the full connection layer, fused and classified with the extracted high-level abstract spatial features, and the *S*^*G*^ is obtained by batch normalization before full connection.

### 3.3. Feature fusion

The deep features extracted from convolution network and graph network are flattened and spliced, as shown below:


(17) $$\begin{array}{l} Output\left(S^{R},S^{G}\right)=Concat\left(flatten\left(S^{R}\right),flatten\left(S^{G}\right)\right) \end{array}$$

Finally, the softmax function is used to output the emotional state. The loss function of this model is the cross-entropy function, and the loss function is minimized using the Adam optimizer with an initial learning rate of 0.0001.

## 4. Experimental results and analysis

### 4.1. Performance analysis

The emotion classification network in the experiment consists of residual network and graph attention neural network. The residual network consists of multiple blocks, and each block contains two convolutional layers. In order to increase the fitting ability of the network, an activation layer is added after all convolutional layers. The first two residual blocks employ 64 filters with a size of 3×3 for convolution calculations, and the last two residual blocks use 128 filters of the same size.

The effect of the proposed network is verified on the DEAP dataset. In order to make the experimental results more objective, 10-fold cross-validation technique is used.

**Figure 5:**
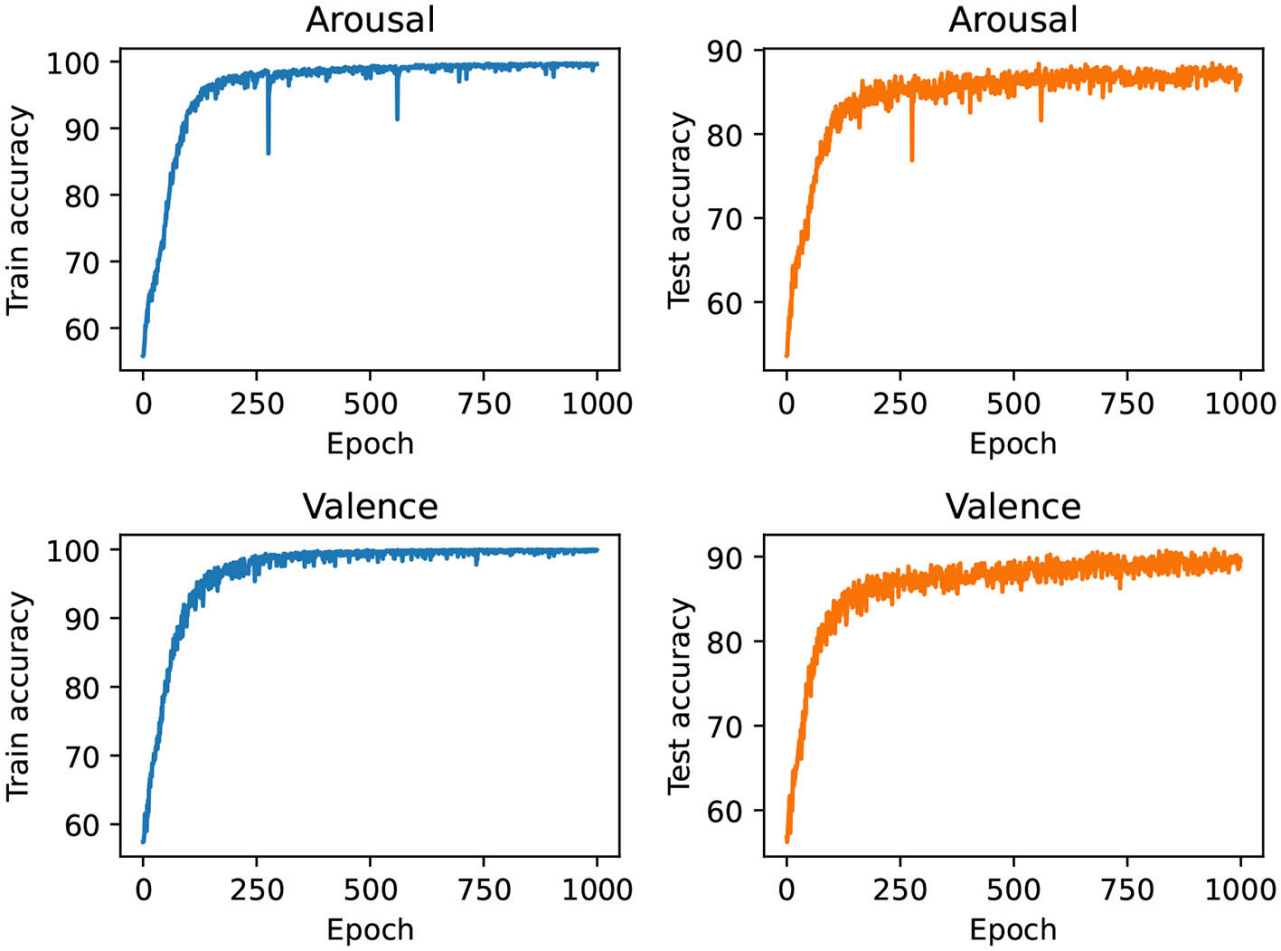
The training process of the proposed network in the two dimensions of Arousal and Valence.

**Figure 6:**
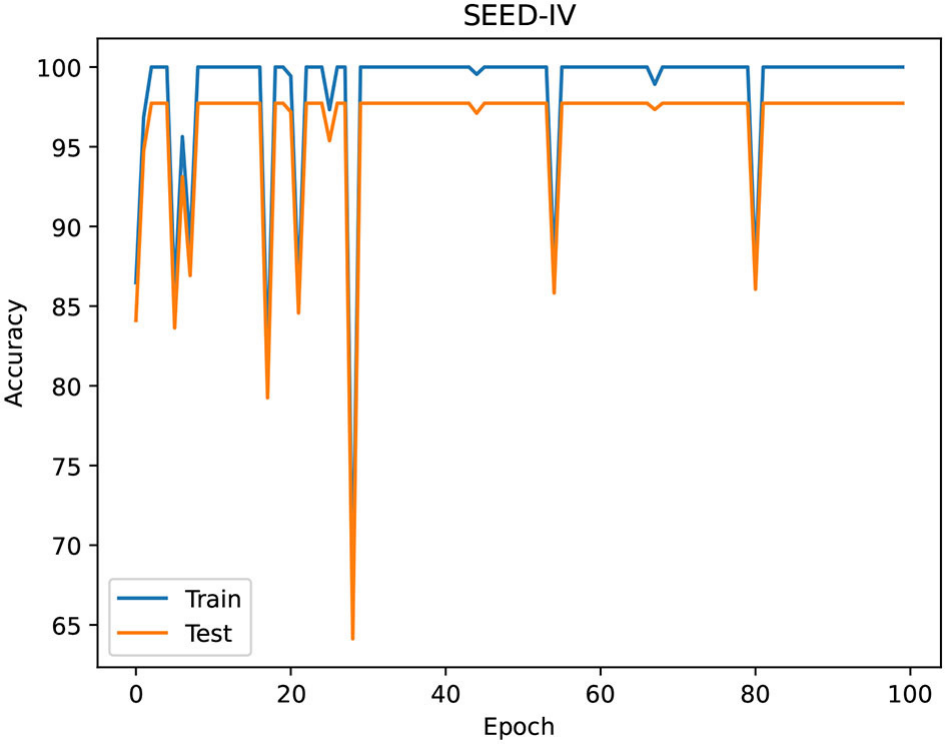
The training process of the proposed network in the SEED-IV.

Figures 5, 6 show the training process of the proposed network on the dataset, Figure 5 shows the training process of the two emotional dimensions of arousal and valence on the DEAP data set, and Figure 6 shows the training process of the SEED data set process. Among them, when the training period is less than 750 in the DEAP dataset, the training accuracy and validation accuracy increase with the increase of the epoch. When the epoch is greater than 750, the training accuracy and validation longitude tend to be stable.

**Figure 7:**
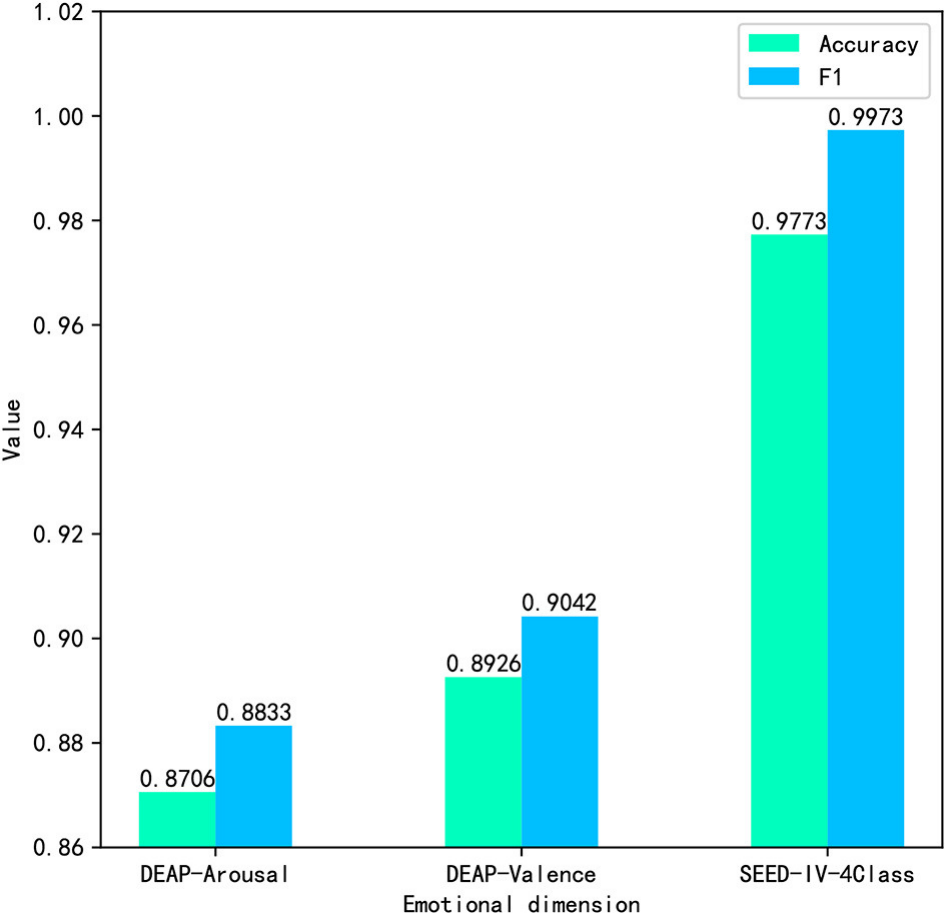
Emotion recognition results of the proposed network for binary classification.

The classification accuracy (Acc) and F1 score (F1) are used to evaluate the performance of the proposed model. The emotion recognition results are shown in Figure 7, respectively. In the arousal dimension, the accuracy is 0.8706 and the F1 score is 0.8833. In the valence dimension, the recognition accuracy and F1 score are 0.8926 and 0.9042, respectively. In addition, 0.9773 Acc and 1.0 F1 were achieved on the four-category task of the SEED-IV dataset. The results of three classified tasks demonstrate the effectiveness of the proposed method.

**Figure 8:**
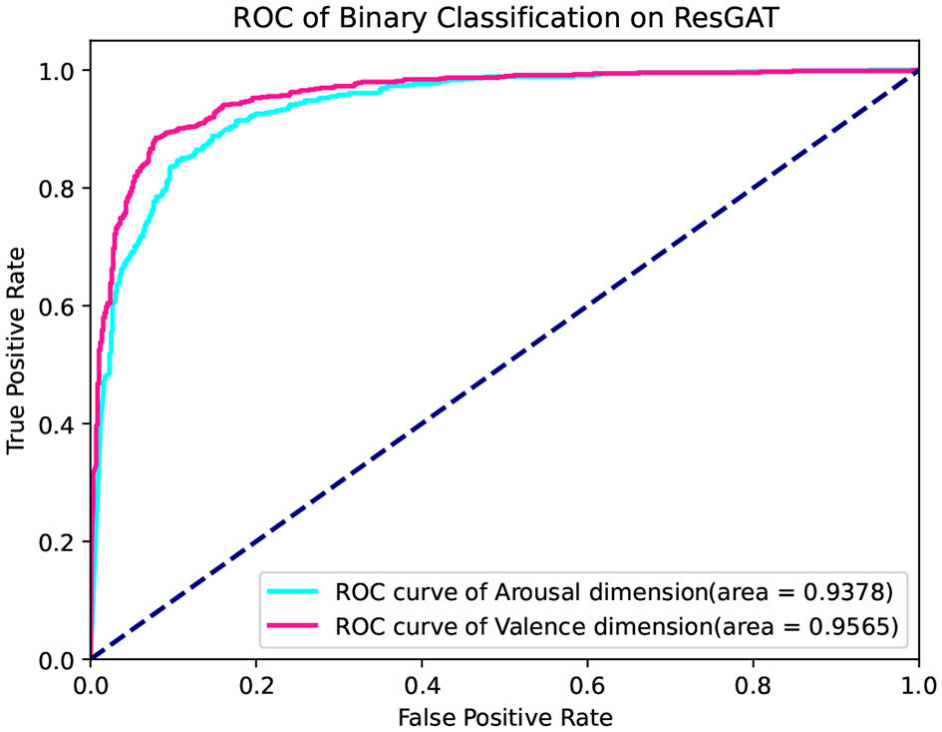
ROC curve of the proposed network.

The receiver operating curve (ROC) is also used to evaluate the performance of the proposed network. The ROC curve is located at the upper left triangle of the square, which reflects a more satisfactory classification rule. The higher the area under ROC Curve (AUC) value, the better the classification effect. Figure 8 shows the receiver operating curves on the two classifications of arousal and valence. The values of AUC in the two dimensions are 0.9378 and 0.9565, respectively. The relatively convex curve and high AUC value prove the excellent classification performance of the proposed classification network.

### 4.2. Comparison between the ensemble method and the single network

In the experiment, an independent GAT model and an independent ResNet model are constructed, respectively. The network structures of the independent GAT and the independent ResNet used in the experiment are consistent with those in the proposed ensemble ResGAT network. When these two independent models are used for emotion classification, the high-level abstract features are flattened and fed into a fully connected layer for classification. The 10-fold cross-validation technique are also used here, and other hyperparameters remain the same.

Firstly, the comparison is carried out on the emotion recognition accuracy. Compared with the GAT model, the proposed ResGAT improves the emotion recognition accuracies by 21.85% in the arousal dimension and 24.68% in the valence dimension. Compared with the ResNet model, the proposed ResGAT improves the emotion recognition accuracies by 1.64% in the arousal dimension and 2.99% in the valence dimension. Secondly, the comparison is carried out on the F1 scores. Compared with the GAT model, the proposed ResGAT improves the emotion recognition accuracies by 17.5% in the arousal dimension and 21.54% in the valence dimension. Compared with the ResNet model, the proposed ResGAT improves the emotion recognition accuracies by 0.9% in the arousal dimension and 2.26% in the valence dimension. The results show that the performance of the proposed ResGAT is obviously better than that of GAT, and it is also improved compared with ResNet.

In addition, it was also verified on the SEED-IV dataset, and the model recognition results are shown in **Table 2**. The experimental results in Tables 1, 2 show that the performance of the integrated network is better than that of a single network, because each network in the integrated network can extract different information, that is, the spatial position information of electrodes and the internal relationship of EEG channels in different brain regions. These two kinds of information complement each other to improve the model recognition performance.

**Table 1:** The results of ResGAT and the two single networks.

**Recognition results**						
**Emotion dimension**	**ResGAT**		**GAT**		**ResNet**	
	**Accuracy**	**F1**	**Accuracy**	**F1**	**Accuracy**	**F1**
Arousal	0.8706	0.8833	0.6521	0.7083	0.8542	0.8743
Valence	0.8926	0.9042	0.6458	0.6888	0.8627	0.8816

**Table 2:** The results of ResGAT and the two single networks (SEED-IV).

**Recognition results**						
**Emotion dimension**	**ResGAT**		**GAT**		**ResNet**	
	**Accuracy**	**F1**	**Accuracy**	**F1**	**Accuracy**	**F1**
Four classification	0.9773	0.9973	0.9522	0.9635	0.9021	0.9251

To further verify the complementarity between the electrode spatial position information and the intrinsic connection relationship between EEG channels, a variety of CNN networks, graph convolution network (GCN) (Kipf and Welling, 2016), vision in transformer network (VIT) (Dosovitskiy et al., 2020) and the ensemble models integrated by the above networks are constructed. The CNN networks constructed specifically include Alex (Krizhevsky et al., 2017), VGGNet (Simonyan and Zisserman, 2014), DenseNet (Huang et al., 2017), and GoogLeNet (Szegedy et al., 2015), which focus on extracting the electrode spatial position information. Similar to GAT, GCN and VIT focus on extracting the intrinsic connection relationship between EEG channels. An ensemble model is constructed by a CNN network and GCN, or by a CNN network and VIT, which means these ensemble models can capture both the electrode spatial position information and the intrinsic connection relationship between EEG channels.

In the CNN networks, the AlexNet structure is affected by the size of the input data. Compared with the structure in the reference (Krizhevsky et al., 2017), the maximum pooling is removed, and the size of the convolution kernel is modified. Other structures remain unchanged. Compared with the residual network in this paper, the structure of VGG only removes the spanning connection. DenseNet employs 1×1 convolutions for better data representation, where the depth of the convolutional layers is 9. GoogLeNet contains a multi-branch convolution structure, which uses convolution kernels of 3×3, 5×5, and 7×7, respectively.

GCN is a natural extension of convolutions on graph structure. Because GCN is suitable for extracting structural features of graphs and can customize local receptive fields, it is widely used in network analysis, traffic prediction. and recommender systems. Inspired by the reference (Kipf and Welling, 2016), a spectral domain-based graph convolutional network is constructed, which contains two convolutional layers. Transformer has been successfully applied in natural language processing and computer vision. Therefore, in the experiment, the standard transformer is directly applied to the EEG signal features with minimal modification. Similar to the reference (Dosovitskiy et al., 2020), the 3D feature matrix is divided into small blocks, and the linear embedding sequence of these blocks is provided as the input of the transformer.

**Figure 9:**
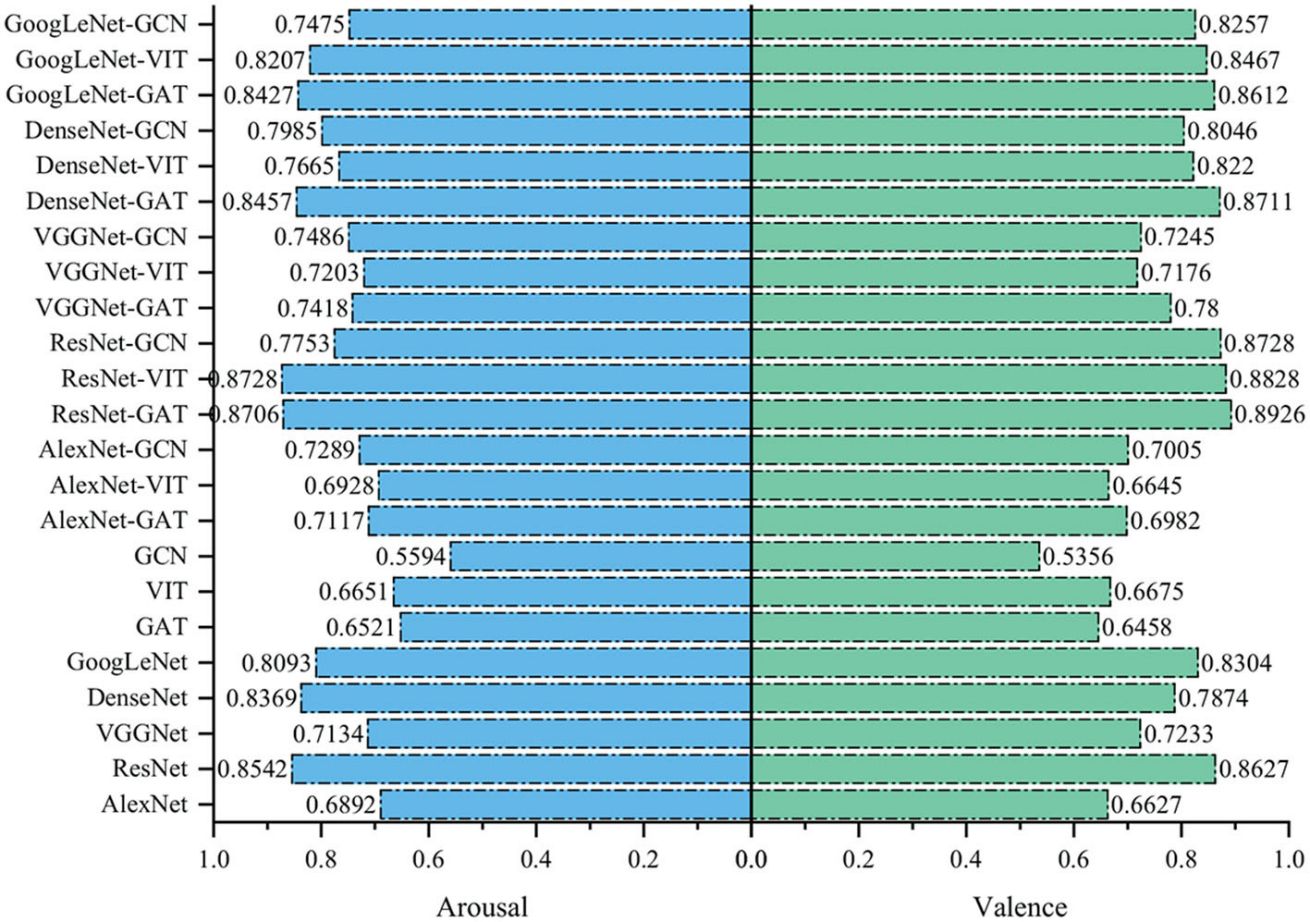
Recognition results of the single networks and the ensemble models integrated by the single networks.

The recognition results of the above networks and the ensemble models are shown in Figure 9. Most of the ensemble models have higher classification accuracy than the corresponding single network, which proves that the electrode spatial position information and the intrinsic connection relationship between EEG channels are complementary to emotion classification. ResNet has the highest classification accuracy in a single network, which achieves 85.42% classification accuracy in arousal dimension and 86.27% classification accuracy invalence dimension, respectively. In the integrated models, ResGAT achieves the highest classification accuracy in the valence dimension, and ResNet-VIT achieves the highest classification accuracy in the arousal dimension. Among the above ensemble models, the ensemble models including GAT performs well in emotion recognition tasks. Combining GAT with any kind of CNN, the classification accuracy can be improved. The experimental results show that GAT has better information capture ability than GCN and VIT, and is more suitable for combining with convolutional networks, which makes the extracted high-level abstract features contain relatively less redundant and irrelevant components.

### 4.3. ResGAT with different model structures

**Figure 10:**
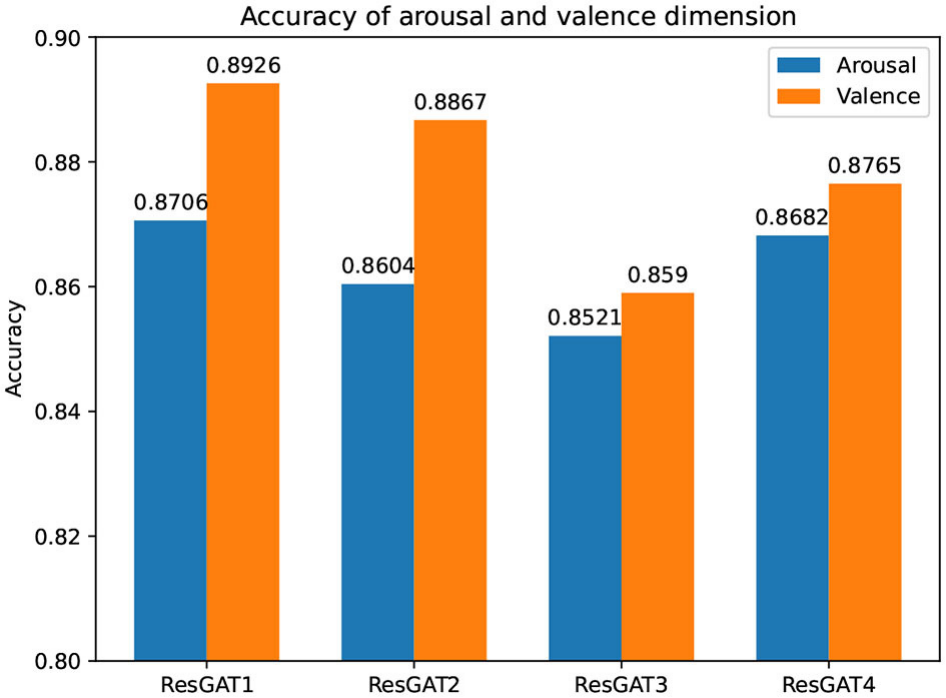
Recognition results of ResGAT1, ResGAT2, ResGAT3, and ResGAT4.

In addition to the ResGAT (ResGAT1) proposed in this paper, three other ResGAT models (ResGAT2, ResGAT3, and ResGAT4) are also constructed. In the ResGAT1, all 3×3 filters are used in the residual network, and the number of multi-head attention in all graph attention layers in GAT is 2. ResGAT2 sets the multi-head attention number of GAT to 4. ResGAT3 is twice as deep as ResGAT1. ResGAT4 uses 3×3 and 5×5 filters to cross the residual structure on the basis of increasing the network depth. The other parameters in ResGAT for the three comparisons remain unchanged. All samples of subjects and 10-fold cross-validation technique are also used here. The recognition results of ResGAT with different structures under the two sentiment annotation schemes are shown in Figure 10.

Compared with ResGAT2, ResGAT3, and ReGAT4, the recognition accuracy of the proposed ResGAT in the arousal dimension is improved by 1.02, 1.85, and 0.24%, respectively. In the dimension of valence, the recognition accuracy of the proposed ResGAT is improved by 0.59, 3.36, and 1.61%, respectively. It can be seen from the comparison results that increasing the complexity and depth of the network will not necessarily improve the accuracy, but will increase the calculation of the model. Therefore, it is very important to choose the appropriate network structure.

### 4.4. Sensitivity analysis

To further prove that the proposed network can extract the spatial domain information of EEG signal channels and learn the internal relationship of different EEG signal channels, the information extraction ability of the proposed model is analyzed.

In the proposed method, the three-dimensional sparse feature matrix and deep residual network are used to capture the dependence between local EEG signal channels. As a contrast, the deep residual network model is also used to deal with the same time-domain characteristics without mapping and arranging according to the international 10/20 standard. Six features of 32 channels can construct a two-dimensional feature matrix with a size of 32×6. The hyperparameters in the experiment remain unchanged. The recognition results using 3D feature matrix and 2D feature matrix, respectively, are shown in Table 3.

**Table 3:** Recognition results using residual networks.

	**3D**	**2D**
DEAP-Arousal	0.8542	0.6203
DEAP-Valence	0.8627	0.6136
SEED-IV	0.9021	0.9020

Compared with the two-dimensional feature matrix, the accuracy of emotion recognition of the three-dimensional feature matrix in the arousal dimension is increased by 23.39%, the accuracy of emotion recognition in the valence dimension is increased by 24.91%, 0.01% improvement on the SEED-IV dataset. The results show that three-dimensional feature matrix and deep residual network can effectively extract local dependency information of signal channels.

**Figure 11:**
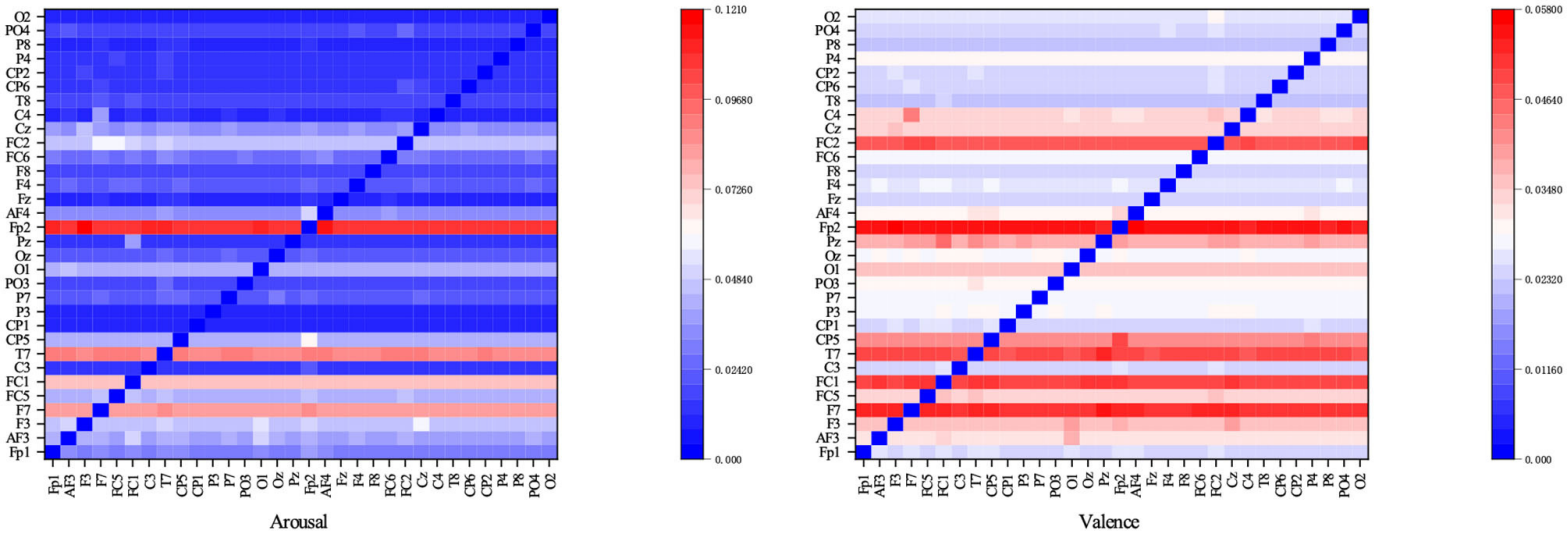
Heatmap representation of adjacency matrices in GAT on DEAP-Arousal and DEAP-Valence affective dimensions.

**Figure 12:**
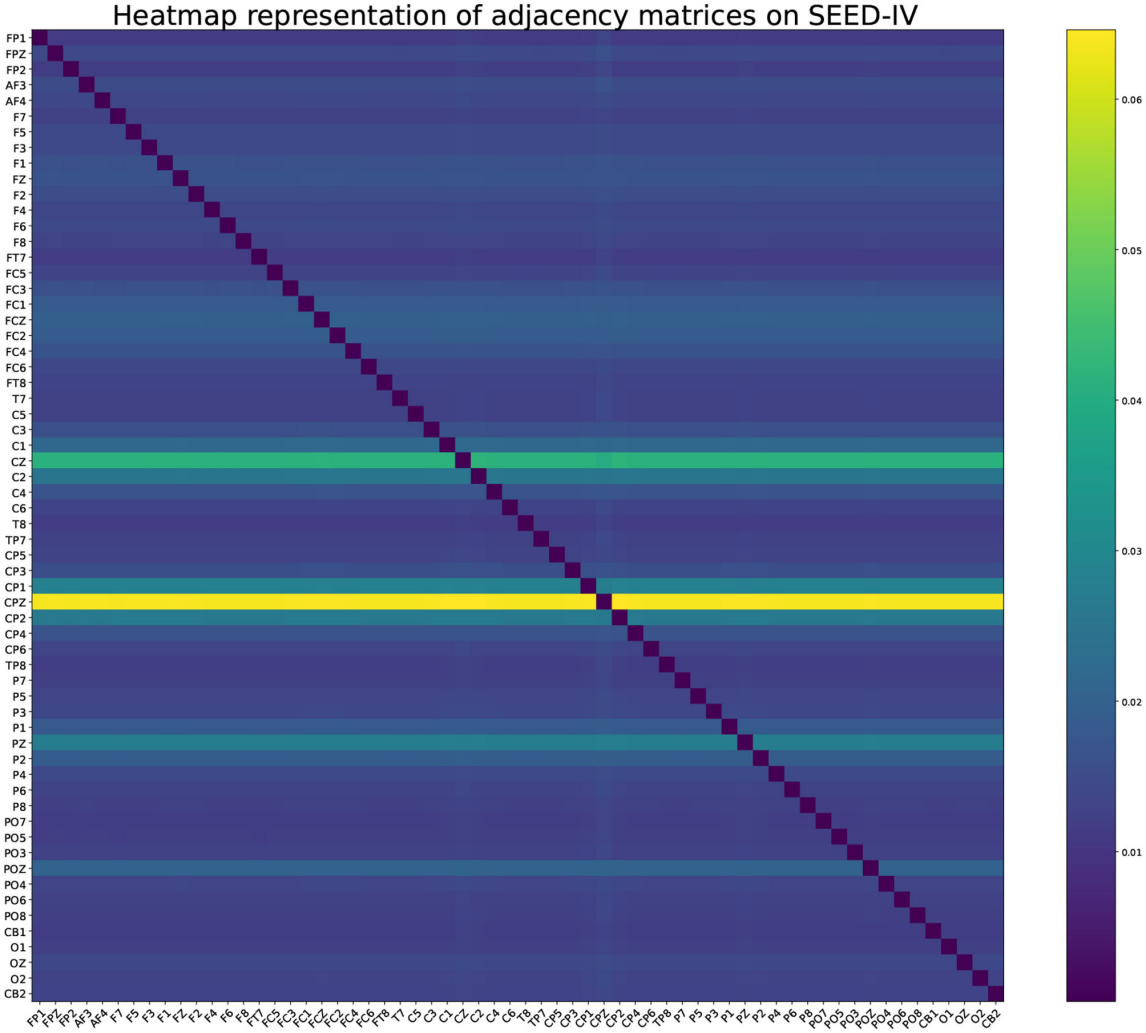
Heatmap representation of adjacency matrices in GAT on SEED-IV.

In order to illustrate the intrinsic connection relationship between EEG channels mined by GAT, the adjacency matrix learned during the training process is displayed. The adjacency matrix is affected by the input data. Input all training data into the GAT in turn to obtain the adjacency matrix corresponding to each sample. The average value of all adjacency matrices can construct a heat map, as shown in Figures 11, 12.

It can be clearly seen that the graph neural network is not limited by distance when collecting neighbor node information in 32 electrode channels. In terms of arousal and valence emotion, C4 electrode channel pays more attention to FC5 channel when aggregating neighbor node information, and FC2 channel pays more attention to F7 and FC5 channels, and CP5 pays more attention to FP2 channel. In the SEED-IV dataset, all nodes focus more on the four channels CZ, CPZ, PZ, and POZ.

### 4.5. t-SNE analysis

**Figure 13:**
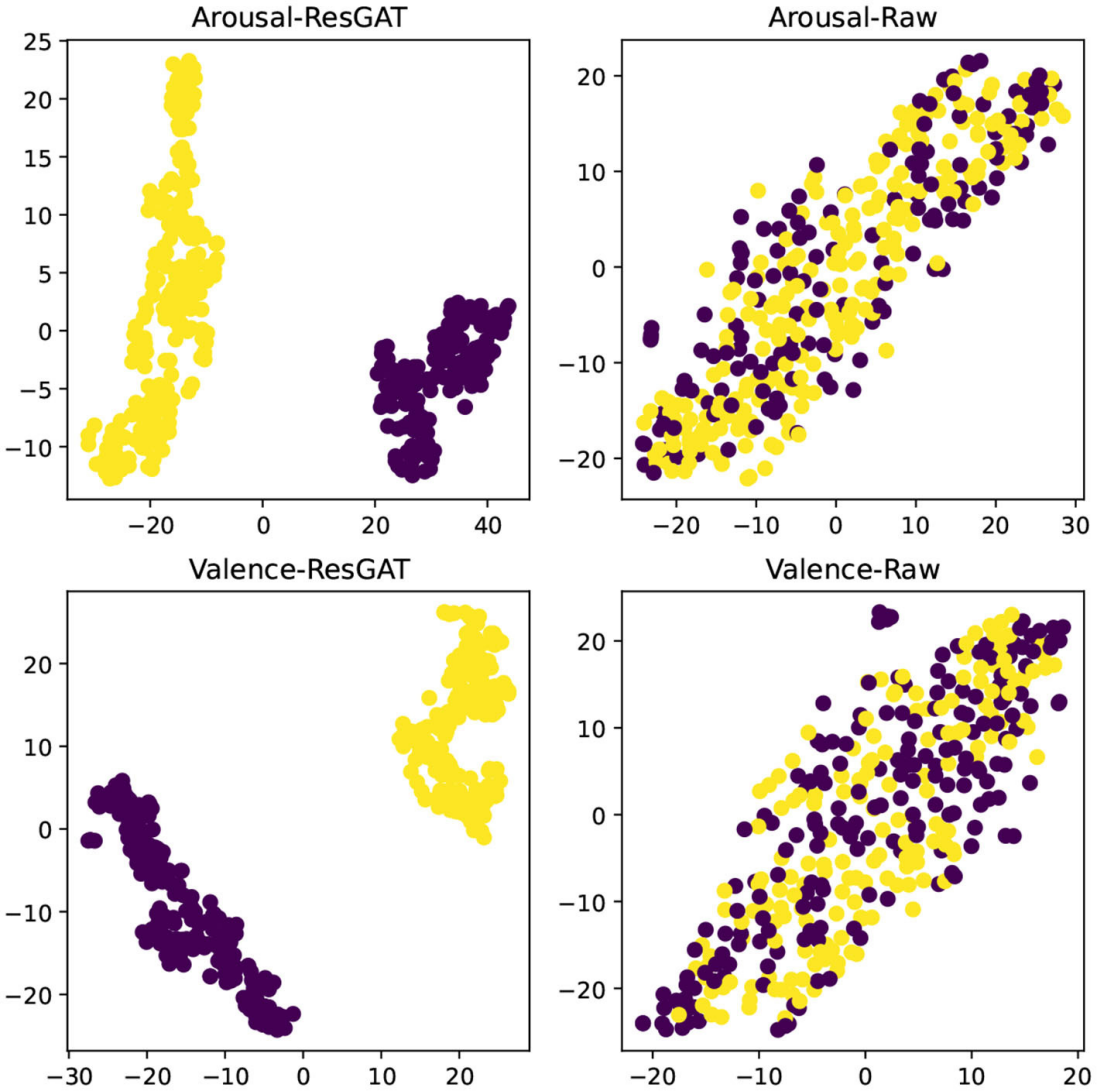
t-SNE analysis on the emotional dimensions of DEAP-Arousal and DEAP-Valence.

**Figure 14:**
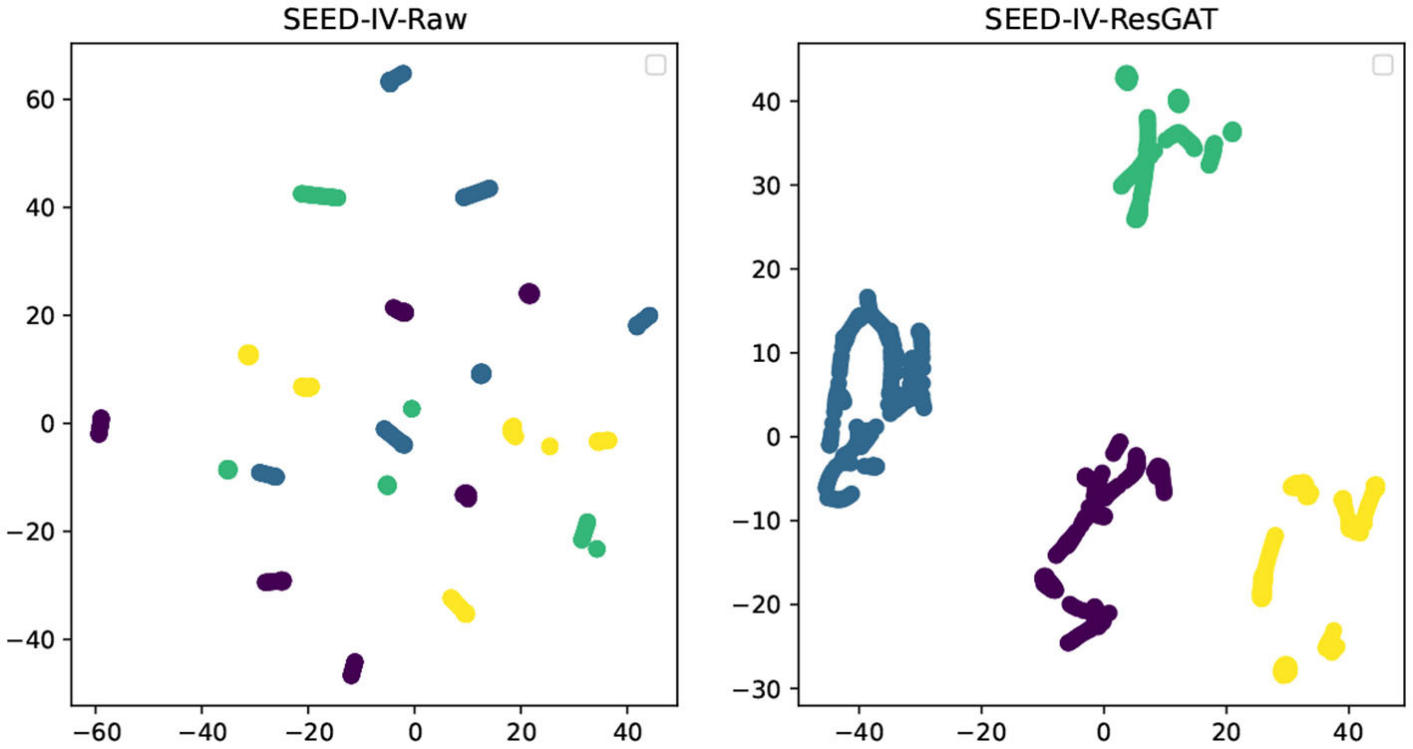
t-SNE analysis on the emotional dimensions of SEED-IV.

In order to demonstrate the effectiveness of ResGAT in extracting high-level abstract features, the t-SNE tool is used to visually analyze the features in two-dimensional space, these features extract all data from a single person. As shown in Figures 13, 14, the input data of the model and the high-level abstract features extracted by ResGAT are displayed in the two emotional dimensions of arousal and valence. The results in the figure demonstrate the effectiveness of the proposed ResGAT in extracting affective state discriminative features.

### 4.6. Comparison with existing methods

The recognition performance of the proposed method is compared with several existing studies. The dataset and labeling scheme are the same for all reported studies. Table 4 details the features and classifiers used in the comparative study. Since recognition accuracy and F1 score are the most commonly used, these two indicators are adopted for comparison. As shown in Table 4, the performance of our approach is better than the comparison methods in both the arousal dimension and the valence dimension. The comparison results show that our approach is excellent in multichannel EEG emotion recognition.

**Table 4:** Details of previous research.

**Study**	**Feature**	**Classifier**	**DEAP**		**SEED-IV**
			**Arousal**	**Valence**	
Samara et al. (2016)	Band power	SVM	0.7367	0.8599	–
Guo et al. (2017)	DWT	SVM	0.6279	0.6021	–
Alhagry et al. (2017)	Raw EEG signals	LSTM	0.8565	0.8545	–
Yang and Liu (2019)	Differential entropy	TCN	0.7140	0.7440	–
Tripathi et al. (2017)	Statistical parameters	DNN	0.7313	0.7578	
		CNN	0.7336	0.8141	0.8599
Gao et al. (2022)	Differential entropy	GCN	0.8193	0.8177	-
Zhong et al. (2020)	Differential entropy	RGNN	-	-	0.7750
Du et al. (2022)	Differential entropy	MD-GCN	-	-	0.9083
Li et al. (2023)	Differential entropy	FGCN	-	-	0.7714
Vafaei et al. (2023)	Time domain features	SAETM	0.8037	0.8173	-
The proposed method	Time domain features	ResGAT	0.8706	0.8926	0.9773

## 5. Conclusion

A novel ensemble deep learning framework is proposed in this work. In the framework, the residual network is employed to extract the spatial position information of the electrode channel through the 3D characteristic matrix. The graph neural network is utilized to learn the neural functional connections between different brain regions, and the multi-head self-attention mechanism is used to adaptively adjust the adjacency matrix in the network. The results show the proposed ResGAT framework makes full use of the electrode spatial position information and the intrinsic connection relationship between EEG channels located in different brain regions. Moreover, the emotion recognition performance of the proposed method is compared with some existing methods and shows advantages, which proves the feasibility and effectiveness of the proposed emotion recognition method.

The experiments in this manuscript were conducted on public datasets DEAP and SEED, and the proposed emotion recognition method demonstrated good performance. However, the number of subjects on the dataset is limited, and the effectiveness of its use in a large population needs further verification. The monitoring and regulation of emotional state is of great significance for the psychological and physiological health of individuals. For example, in clinical treatment, monitoring and regulating emotional states can help doctors better understand patients' emotional states, thereby providing more personalized and effective treatment plans for patients. In daily life, monitoring and regulating emotional states can help individuals better manage their emotions and improve their quality of life. Moreover, the emotion recognition performance of the proposed method is compared with some existing methods and shows advantages, which proves the feasibility and effectiveness of the proposed emotion recognition method. In addition, the proposed emotion recognition classification model can also be applied in disease diagnosis, such as identification of patients with depression; issuing execution commands to control external devices, helping patients to carry out active rehabilitation training; diagnosis of schizophrenia; quantifying the neurophysiological changes associated with a variety of work-related physical activities (Ismail et al., 2023).

## Data availability statement

Publicly available datasets were analyzed in this study. This data can be found here: http://www.eecs.qmul.ac.uk/mmv/datasets/deap/.

## Author contributions

HC designed and organized the study and wrote this article. HC, YL, and YC collected and analyzed the data. All authors contributed to manuscript revision, read, and approved the submitted version.
