# Corrigendum: Multi-channel EEG emotion recognition using Residual Graph Attention Neural Network

**DOI:** 10.3389/fnins.2023.1283644

**Published:** 2023-11-28

**Authors:** Hao Chao, Yiming Cao, Yongli Liu

**Affiliations:** College of Computer Science and Technology, Henan Polytechnic University, Jiaozuo, China

**Keywords:** EEG, emotion recognition, residual network, graph attention neural network, feature fusion

In the published article, there was an error in the Funding statement. The fund name is incorrect and the fund number is missing. The original text is: Programs for Science and Technology Development of Henan province. The correct Funding statement appears below.

## Funding

Programs for Science and Technology Development of Henan province No. 222102210078.

The authors apologize for this error and state that this does not change the scientific conclusions of the article in any way. The original article has been updated.

